# Intraocular and serum cytokine profiles in patients with intermediate uveitis

**Published:** 2011-07-20

**Authors:** Natasa Vidovic Valentincic, Jolanda D.F. de Groot-Mijnes, Aleksandra Kraut, Peter Korosec, Marko Hawlina, Aniki Rothova

**Affiliations:** 1 Eye Hospital, University Medical Centre, Ljubljana, Slovenia; 2 University Hospital for Pulmonary and Allergic diseases, Golnik, Slovenia; 3 Department of Virology, University Medical Center Utrecht, The Netherlands; 4 Department of Ophthalmology, University Medical Center Utrecht, The Netherlands

## Abstract

**Purpose:**

To study the intraocular and serum cytokine and chemokine profile in patients with intermediate uveitis (IU) at various stages of inflammatory activity.

**Methods:**

Institutional, prospective association study. Paired aqueous humor (AqH) and serum samples were collected from 36 consecutive IU patients and 10 controls. The concentrations of interleukin (IL)-1β, IL-6, IL-8, IL-10, IL-12p70, tumor necrosis factor (TNF)-α, CC - chemokine ligand 5/regulated upon activation normal T-cell expressed, and secreted (CCL5/RANTES), CC - chemokine ligand 3/macrophage inflammatory protein 1alpha (CCL3/MIP-1α), CCL4/MIP-1β, and CC - chemokine ligand 2/monocyte chemotactic protein - 1 (CCL2/MCP-1) were measured in both AqH and serum by multiplex immunoassay. Main outcome measures were serum and intraocular levels of the analyzed cyto- and chemokines.

**Results:**

Patients with IU had higher serum levels of TNF-α than non-uveitic controls (p<0.0001), whereas their AqH TNF-α levels did not show a difference (p=0.323). IU patients had higher intraocular levels of IL-1β, IL-6, IL-8, IL-10, IL-12p70 and CCL2/MCP-1 than the controls (p=0.020, 0.001, <0.0001, 0.005, 0.003, and 0.003, respectively). Active stages of IU were characterized by higher levels of IL-6, IL-8, CCL5/RANTES and CCL2/MCP-1 (p=0.003, <0.0001, 0.033, and 0.033, respectively). Higher levels of IL-6 and IL-8 were found in IU patients with cystoid macular edema (CME) compared to non-CME IU patients (p=0.026 and 0.012, respectively). Significant positive correlations between various observed mediators were present in the AqH of IU patients only.

**Conclusions:**

Significantly elevated concentrations of multiple intraocular cytokines were found in IU patients, especially IL-6 and IL-8 in those with CME and active disease. In serum elevated TNF-α levels were observed in IU patients. Our findings improve the understanding of the pathogenesis of IU and contribute to the identification of factors which may contribute to the activity of IU.

## Introduction

Intermediate uveitis (IU) represents a chronic type of uveitis which usually manifests at young adult age with the vitreous and peripheral retina as the major sites of inflammation. The cause and pathogenesis of IU are not known. The associated systemic diseases include mostly multiple sclerosis (MS) and sarcoidosis; however the majority of cases are idiopathic [1]. It is not clear whether the inflammation in idiopathic cases is limited solely to the eye or whether there is (subclinical) inflammatory activity elsewhere in the body.

Cytokines and chemokines play a major role in the pathogenesis and persistence of intraocular inflammation [2]. In this prospective study we compared the intraocular and serum cytokine profiles in 36 patients with IU and related the laboratory results to clinical features and various stages of inflammatory activity.

## Methods

This was an institutional, prospective association study performed between 2007 and 2009. Paired aqueous humor (AqH) and serum samples were collected from 36 consecutive IU patients in various stages of inflammatory activity and from 10 control patients with cataract and no uveitis. AqH and serum samples were collected from the control patients during cataract surgery.

AqH sampling was done with a standardized procedure described by van der Lelij et al. [3]. In our uveitis patients AqH samples were taken with the help of a head magnifying lens while the patient lay supine on an operating chair. A lid speculum was used to spread the eyelids. Local anesthesia was given with Alcaine (proparacaine hydrocloride 0.5%; Alcon, Fort Worth, TX).The ocular surface was sterilized with povidone iodine and irrigated with 0.9% NaCl. The eye was fixated firmly at limbus with Fluid Analysis Set (FAS) tweezers (L.KLEIN AG, Biel, Switzerland). A corneal pre-incision was made and up to 200 µl of AqH was aspirated with a 27 gauge tuberculin syringe. Ten control AqH samples were obtained during cataract surgery before the initial incision was made. Samples were stored immediately at −80 °C in sterile screw-cap tubes and thawed immediately before analysis.

Patients with IU were selected from a database of uveitis patients from the Eye Hospital of the University Medical Centre in Ljubljana, Slovenia. Our patients with IU represented a consecutive series of patients who visited our clinic between 2007 and 2009 and were subjected to AqH analysis. The study protocol was approved by the local ethics committee at the Ministry of Health, Republic of Slovenia, and signed informed consent was obtained from each patient. The study was performed in accordance with the Declaration of Helsinki.

The diagnosis of IU was made according to the diagnostic criteria of the standardization of uveitis nomenclature for Reporting Clinical Data [4]. There were 36 patients with a male-to-female ratio of 1.0:1.1. Mean age at onset of IU was 38 years, median age at sampling was 44 years. Fifteen patients had quiescent IU and 21 patients had active IU at the time of sampling. Quiescence of IU was defined as the absence of active inflammation (with the exception of sporadic cells in the vitreous) together with the absence of cystoid macular edema (CME), snowballs, and snow banking. In addition, to be classified as quiescent IU, patients had not received systemic treatment or periocular injections for at least 1 year. Seven patients had CME. Our series included 5 patients with multiple sclerosis, 3 patients with sarcoidosis and 1 with Lyme disease. During the sampling, 10 patients were on systemic therapy, but none of them had received anti-tumor necrosis factor (anti-TNF) treatment.

The concentration of inflammatory mediators was measured by the Cytometric Bead Arrays method (BD Biosciences, San Diego, CA) and included the measurement of interleukin (IL)-1β, IL-6, IL-8, IL-10, IL-12p70, TNF-α, Chemokine (C-C motif) ligand 5, also known as Regulated upon Activation, Normal T-cell Expressed and Secreted (CCL5/RANTES), CCL3/ Macrophage Inflammatory Protein −1α (MIP-1α), CCL4/MIP-1β, and CCL2/MCP-1 in paired AqH and serum of all subjects.

AqH (50 µl) and 50 µl of serum were used for cytokine analysis. Flow cytometry was performed using a FACSCalibur flow cytometer (Becton, Dickinson and Company, Frankling Lakes, MD). Data were acquired and analyzed using Cytometric Bead Array (CBA; Becton, Dickinson and Company) software. Concentrations above or below the detection limit were given as the highest or lowest detectable value. For statistical analysis concentrations below the detection limit were converted to a value of 0.5×, the lowest point of the calibration curve [5].

SPSS 15.0.1 for Windows (SPSS Inc., Chicago, IL) was used for statistical analysis. The Mann–Whitney *U-*test was used for nonparametric comparison of the geometric means of the different groups. For analysis of correlations between inflammatory mediators the Spearman's Rho test was used. A p value of <0.05 was considered statistically significant.

## Results

### Serum cytokine and chemokine levels

**Figure 1:**
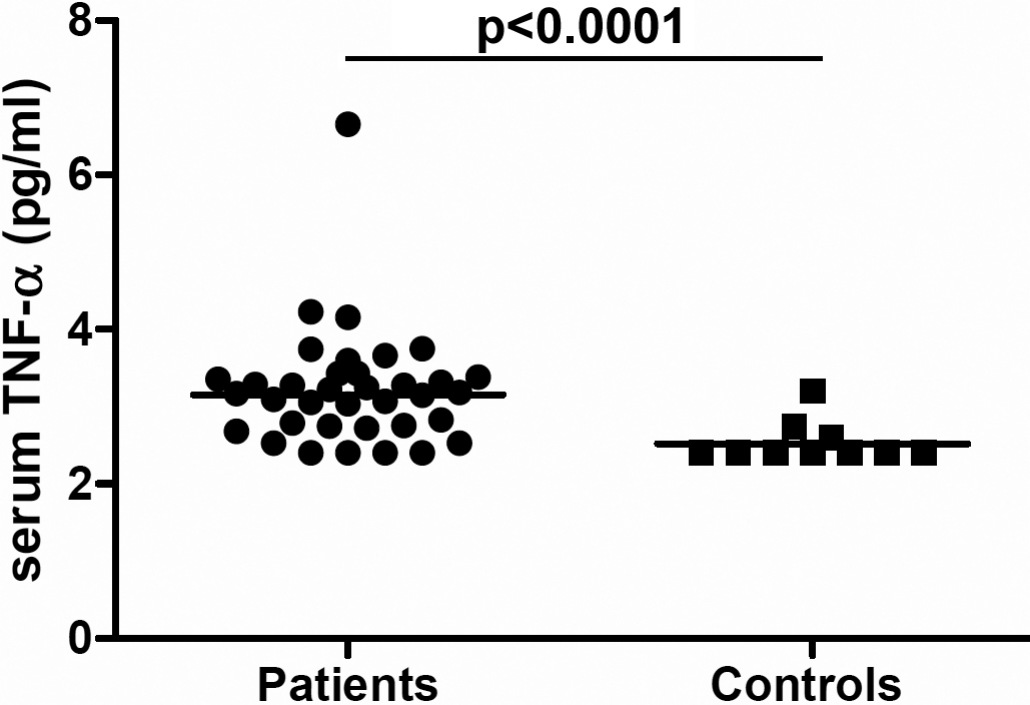
Dotplot graph showing serum concentrations of TNF-α in all patients with IU and in the control group. Statistical significance is indicated at the top (Mann–Whitney *U*-test). The horizontal line represents the geometric mean.

Serum cytokine and chemokine levels are given in Table 1. Serum TNF-α levels were significantly higher in IU patients than in controls (p<0.0001, Figure 1). No other differences in serum cytokine levels between IU and non-uveitic controls were noted. Serum cytokine and chemokine levels did not differ between active and non-active IU patients, between IU patients with and without CME, and between the IU patients with or without systemic disease. However, patients on systemic therapy had lower serum levels of TNF-α than patients who were not on treatment (p=0.034).

**Table 1:** Serum cytokine levels in patients with intermediate uveitis and in controls.

**Inflammatory mediators**	**Controls (n=10)**	**IU patients (n=36)**	**Active IU (n=15)**	**Quiescent IU (n=21)**	**CME (n=7)**	**No CME (n=29)**	**Systemic therapy (n=10)**	**No systemic therapy (n=26)**	**Systemic disease (n=9)**	**No systemic disease (n=27)**
Mean age (yrs)	40	36	42	32	44	35	43	36	44	34
Geometric mean (pg/ml) Median (pg/ml) Range (pg/ml) p value										
IL-1β (2.0)*	4.0 4.8 2.0–6.8	2.9 2.9 2.0–11.7	2.8 3.2 2.0–11.7	2.5 2.6 2.0–5.7	1.8 1.1 2.0–4.4	2.9 3.5 2.0–11.7	1.9 3.2 2.2–6.7	2.9 4.0 2.0–11.7	1.5 1.1 2.0–4.5	3.1 3.6 2.0–11.7
	0.053		0.820		0.110		0.101		0.127	
IL-6 (0.5)	3.6 3.3 2.6–8.1	3.3 3.3 2.4–17.4	4.0 3.4 2.6–17.4	3.6 3.3 2.4–6.8	4.8 4.0 3.1–17.4	3.5 3.2 2.4–7.5	4.1 4.0 2.7–7.5	3.6 3.2 2.4–17.4	4.3 4.0 3.1–6.8	3.6 3.2 2.4–17.4
	0.990		0.657		0.121		0.063		0.693	
IL-8 (0.4)	8.6 8.7 2.3–33.3	8.4 8.4 3.7–14.3	8.3 8.5 4.6–13.4	8.0 7.4 3.7–14.3	8.5 8.4 6.1–11.5	8.0 8.3 3.7–14.3	8.7 8.9 4.6–14.3	7.9 7.4 3.7–13.4	9.0 9.1 6.8–11.5	7.8 8.0 3.7–14.3
	0.783		1.000		0.930		0.566		0.180	
IL-10 (1.5)	2.5 2.3 1.5–9.9	2.1 2.1 1.5–7.0	2.2 2.3 1.5–7.0	1.9 2.1 1.5–3.6	2.6 2.9 1.5–7.0	1.9 2.1 1.5–5.4	2.5 2.4 1.5–7.0	1.9 2.0 1.5–5.4	1.7 1.9 1.5–2.9	2.1 2.2 1.5–7.0
	0.286		0.202		0.065		0.256		0.192	
TNF-α (2.0)	1.6 1.3 2.0–3.2	3.2 3.2 2.0–6.7	2.8 2.8 2.0–6.7	3.0 3.3 2.0–4.2	3.0 3.1 2.5–4.2	2.9 3.3 2.0–6.7	2.5 2.9 2.0–6.7	3.1 3.3 2.0–6.7	2.7 3.1 2.0–3.3	3.0 3.3 2.0–6.7
	**<0.0001**		0.072		0.387		**0.034**		0.157	
IL-12p70 (1.5)	1.9 2.2 1.5–2.7	2.2 2.2 1.5–3.8	1.8 2.2 1.5–3.7	2.1 2.2 1.5–3.8	1.4 2.2 1.5–3.2	2.1 2.2 1.5–3.8	1.5 1.5 1.5–3.8	2.1 2.2 1.5–3.7	1.7 1.9 1.5–3.5	2.0 2.2 1.5–3.8
	0.990		0.401		0.206		0.135		0.349	

*: lower detection limit for each cytokine (pg/ml). IU: intermediate uveitis; CME: cystoid macular edema.

### Aqueous humor cytokine and chemokine levels

**Figure 2:**
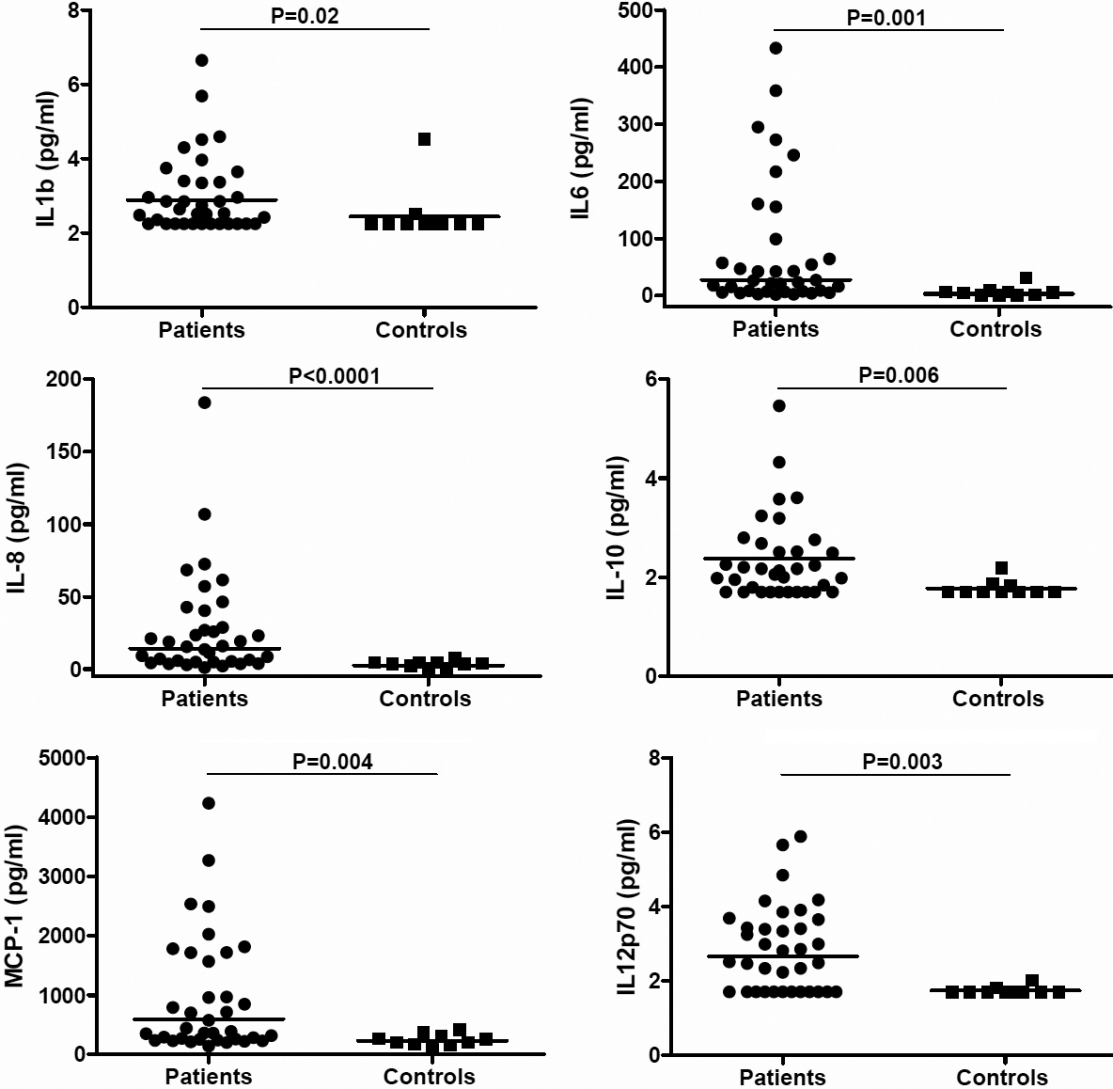
Dotplot graphs showing aqueous humor concentrations of IL-1β, IL-6, IL-8, IL-10, IL-12p70, and CCL2/MCP-1 in all patients with IU and in the controls. Statistical significances are indicated at the top (Mann–Whitney *U-*test). The horizontal lines represent the geometric means.

Intraocular cytokine levels of patients with IU and controls are given in Table 2. In most IU patients the cytokine and chemokine levels were higher in the ocular fluids than in the corresponding sera; the levels of the two most abundant mediators, IL-6 and IL-8, exceeded those of serum in 23/26 (88%; p<0.0001) and 24/36 (66%; p=0.038), respectively. In the controls, the intraocular levels of IL-1β, IL-8, IL-10 and IL-12p70 were lower than in serum. Intraocular samples of IU patients had significantly higher levels of IL-1β (p=0.02), IL-6 (p=0.001), IL-8 (p<0.001), IL-10 (p=0.005), IL-12p70 (p=0.003), and CCL2/MCP-1 (p=0.003) compared to the controls (Figure 2). No differences in intraocular cytokines levels were noted between patients with IU-associated systemic disease and idiopathic IU.

**Table 2:** Aqueous humor cytokine levels in patients with intermediate uveitis and in controls.

**Inflammatory mediators**	**Controls (n=10)**	**IU patients (n=36)**	**Active disease (n=15)**	**Quiescent disease (n=21)**	**CME (n=7)**	**No CME (n=29)**	**Systemic therapy (n=10)**	**No systemic therapy (n=26)**	**Systemic disease (n=9)**	**No systemic disease (n=27)**
Mean age (yrs)	40	36	42	32	44	35	43	36	44	34
Geometric mean (pg/ml) Median (pg/ml) Range (pg/ml) p value										
IL-1β (2.0)*	1.4 1.1 2.2–4.5	2.3 2.5 2.2–6.7	2.1 2.1 2.2–6.7	2.5 2.2 2.2–4.6	2.5 2.5 2.2–6.7	2.2 2.6 2.2–5.7	2.3 2.5 2.2–6.7	2.3 2.7 2.2–5.7	2.5 4.0 2.2–4.5	2.2 3.2 2.2–6.6
	**0.020**		0.279		0.845		0.876		0.641	
IL-6 (0.5)	3.3 4.8 0.6–31.0	27.5 24.5 2.0–433.1	66.5 57.3 5.6–433.1	14.6 15.4 2.1–160.6	86.7 64.4 5.6–433.1	20.8 20.7 2.0–294.0	40.1 34.6 5.6–358.4	23.8 21.8 2.1–433.1	32.9 42.6 2.0–295.0	25.9 21.5 2.3–433.1
	**0.001**		**0.003**		**0.026**		0.393		0.693	
IL-8 (0.4)	2.6 3.8 0.4–7.9	14.2 15.6 1.0–84.0	34.0 25.8 9.2–183.9	7.6 5.9 1.0–57.1	40.4 25.8 15.5–83.9	11.0 9.2 1.0–106.7	26.2 24.4 3.8–183.9	11.2 10.3 1.0–106.7	17.6 26.8 2.9–68.4	13.2 13.6 1.0–183.9
	**<0.0001**		**<0.0001**		**0.012**		0.074		0.565	
IL-10 (1.5)	1.1 2.6 1.7–2.2	2.0 2.8 1.7–14.7	2.5 3.0 1.7–14.7	1.7 2.5 1.7–5.5	2.5 2.8 1.7–4.3	1.9 2.0 1.7–14.7	2.1 2.6 1.7–3.6	1.9 2.0 1.7–14.7	2.1 2.5 1.7–5.5	2.0 2.1 1.7–14.7
	**0.005**		0.062		0.054		0.256		0.641	
TNF-α (2.0)	2.0 2.8 2.4–3.3	2.4 2.6 2.4–5.4	2.7 2.8 2.4–5.2	2.2 2.8 2.4–5.4	3.1 3.3 2.4–5.2	2.2 2.7 2.4–5.4	3.2 3.0 2.4–5.2	2.9 2.6 2.4–5.3	2.3 2.7 2.4–5.2	2.3 3.0 2.4–5.4
	0.323		0.462		0.094		0.135		0.247	
IL-12p70 (1.5)	1.0 0.9 1.7–2.0	2.1 2.7 1.7–5.9	2.1 2.5 1.7–5.9	2.2 3.0 1.7–4.8	2.8 2.5 1.7–5.9	2.0 2.8 1.7–4.8	2.4 3.0 1.7–5.9	2.1 2.5 1.7–4.8	2.5 3.0 1.7–4.8	2.0 2.5 1.7–5.9
	**0.003**		0.505		0.480		0.520		0.565	
CCL5/RANTES (0.02)	1.8 1.5 1.1–3.9	3.6 2.3 0.0–76.5	6.3 3.7 0.5–59.4	2.1 2.0 0.0–76.5	8.4 8.5 2.0–59.4	2.9 2.1 0.0–76.5	4.5 5.7 0.0–43.0	3.3 2.1 0.5–76.5	8.9 8.5 0.5–76.5	2.7 2.3 0.0–59.4
	0.091		**0.033**		0.078		0.355		0.192	
CCL3/MIP-1α (4.0)	3.2 3.1 4.1–9.1	4.0 4.9 4.1–15.2	4.5 5.3 4.1–8.0	3.7 4.8 4.1–15.3	3.9 4.2 4.1–8.0	4.1 5.0 4.1–15.3	4.2 4.7 4.1–8.0	4.0 5.2 4.1–15.3	5.0 5.0 4.1–15.3	3.8 4.8 4.1–8.0
	0.812		0.202		0.907		0.931		0.368	
CCL4/MIP-1β (3.0)	27.9 24.3 19.0–43.8	34.6 32.3 16.4–190.9	35.5 34.4 16.4–85.0	34.0 30.2 16.9–190.4	33.8 34.4 16.4–85.0	34.8 32.0 16.9–190.4	34.5 32.3 16.4–190.4	35.1 31.8 18.4–85.0	39.7 32.7 16.9–190.4	33.1 30.5 16.4–85.0
	0.350		0.657		0.876		0.821		0.720	
CCL2/MCP1 (3.0)	235.9 232.4 140.3–415.5	595.7 417.5 143.4–4230.1	888.6 846.7 214.6–3270.0	447.7 316.9 143.1–4230.1	857.4 846.7 222.7–2536.0	545.6 361.0 143.1–4230.1	767.9 781.0 214.6–3270.0	540.3 356.3 143.1–4230.1	901.0 959.9 143.1–4230.1	519.0 363.9 200.1–3270.0
	**0.003**		**0.033**		0.236		0.320		0.157	

*: lower detection limit for each cytokine (pg/ml). IU: intermediate uveitis; CME: cystoid macular edema.

Active IU was characterized by higher levels of intraocular IL-6 (p=0.003), IL-8 (p<0.0001), CCL5/RANTES (p=0.033), and CCL2/MCP-1 (p=0.033) compared to IU in remission; borderline significance was observed for IL-10 (p=0.062). No significant associations were found for the other measured cytokines (Table 2). The IU patients on systemic therapy had similar levels of intraocular cytokines as the IU patients who were without systemic treatment; in particular no differences were noted for intraocular TNF-α levels. The presence of CME in IU patients was associated with higher intraocular levels of IL-6 and IL-8 (p=0.026 and p=0.012, respectively), whereas a borderline difference was observed for IL-10 (p=0.053).

### Correlations

No significant correlations of cytokines and chemokines were observed between the AqH and the serum . However, significant positive correlations were observed in the AqH of IU patients between IL-6 and IL-8 (p=<0.0001, *r*=0.8), IL-1β and IL-12p70 (p<0.001, *r*=0.7), IL12p70 and TNF-α (p<0.001, *r*=0.6), IL-10 and TNF-α (p<0.0001, *r*=0.7), and between RANTES and IL-6 (p=0.001, *r*=0.6) and IL-8 (p<0.0001, *r*=0.6), respectively. No significant correlations could be detected for other analyzed cytokines and chemokines in serum of IU and control patients.

## Discussion

The most striking findings of our study were the highly elevated levels of intraocular IL-6 and IL-8 and the elevated serum TNF-α level in IU patients. Increased serum TNF-α level was also present in patients with idiopathic IU and no apparent systemic disorder. Serum TNF-α levels were lower in IU patients on systemic treatment, but their intraocular TNF-α levels were not increased and the levels of diverse cytokines and chemokines in their intraocular fluids did not correlate with their serum levels. The presence of active inflammation and CME was characterized by elevated intraocular levels of IL-6 and IL-8.

TNF-α is a crucial cytokine involved in inflammatory processes. It is a proinflammatory cytokine, important for the induction and maintenance of inflammation in autoimmune reactions [6,7]. TNF-α is released from macrophages and T cells during inflammatory responses, influences leukocyte activation and infiltration via adhesion molecule upregulation and macrophage activation and drives Th1 T lymphocyte responses within tissues [8]. The high TNF-α levels observed in the serum of our IU patients are in accord with earlier findings of high expression of TNF-α by peripheral blood CD4+ lymphocytes in patients with active uveitis of different origins, in active Behçet disease and in presumed ocular sarcoidosis [9-11]. In contrast, the TNF-α levels in the AqH of patients with active idiopathic uveitis were reported to be within normal limits, which is consistent with our results on IU. These findings imply that systemic participation of TNF-α might be more important than such participation in a local environment. We observed lower levels of TNF-α in the serum of IU patients on systemic therapy although anti-TNF drugs were not administered. A possible explanation could be that systemic immunosuppressive drugs reduce inflammation and inhibit cells that produce TNF-α.

IL-6 is a cytokine with pleiotropic functions. It is important in the development of Th 17 cells and is a major regulator of the acute phase response [12]. IL-8 is a proinflammatory cytokine, which has profound effects on neutrophils and chemoattraction of T lymphocytes, and can induce surface expression of adhesion molecules [13-15]. IL-8 also enhances the migration of neutrophils and CCL2/MCP-1 toward monocyte/macrophage infiltrations [12]. IL-6 and IL-8 are major proinflammatory cytokines in uveitis and elevated intraocular levels were found repeatedly in the intraocular fluids of patients with uveitis of diverse origins, including ocular toxoplasmosis, viral uveitis, Fuchs heterochromic uveitis syndrome (FHUS) and Behçet’s disease as well as in ocular fluids of children with uveitis [16-18]. Apparently, IL-6 and IL-8 are general markers of active uveitis and are not specific for particular uveitis entities. The triad of high intraocular IL-8, IL-6 and CCL2/MCP-1 levels was previously reported in patients with chronic endogenous uveitis [12,17,19]. A strong correlation between IL-6 and IL-8 was noted, which agrees with our findings [12].

Elevated IL-8 serum levels were found in a study of 61 patients with untreated and active IU and in patients with active noninfectious uveitis [20,21]. Our results are entirely different and none of our IU patients had serum IL-8 levels above 20 pg/ml. These differences might be explained by the fact that the samples in our study originated from later stages in the disease process.

IL-10 regulates differentiation and proliferation of multiple immune cells such as T and B cells, antigen-presenting cells and granulocytes. It controls the inflammatory processes by suppressing the expression of proinflammatory cytokines such as TNF-α, IFN-γ and IL-1β [22,23]. Elevated intraocular IL-10 levels were previously associated with activity of uveitis and it was hypothesized that elevated levels of IL-10 represent an attempt to control the inflammation [4,24-26]. IL-12 is a dominant factor in the development of Th1 cell responses [27]. The intraocular levels of IL-10 and IL-12 in uveitis reported in the literature are controversial [28,29]. Although intraocular levels of IL-10 and IL-12 were higher in IU than in controls, these were not associated with IU activity or with CME which is in agreement with previous observations [24,30].

Reports on intraocular cytokines in inflammatory CME are inconsistent and so far no specific cytokine profile characteristic for CME was observed. We found significantly higher levels of IL-6 and IL-8 in patients with CME, which differs from the results from an earlier study on patients with uveitis of various origins [26]. Moreover, we observed no significant associations between the presence of systemic disease and levels of specific cytokines either in serum or intraocular fluids. This may be explained either by the limited number of included patients or by the fact that cytokine production in the eye differs from cytokine production in the peripheral blood and the observation that the activity of intraocular inflammation does not necessarily parallel the activity of systemic disease.

In most studies, including the present series, the analyses of intraocular cytokines were performed at various stages of the disease processes and with various types of treatments already having been employed, which could obviously have influenced the results obtained. Lower intraocular levels of IL-8 and IL-10 were observed in AqH of children with uveitis treated with systemic methotrexate, but none of our patients used methotrexate at the time of sampling [5].

Longitudinal measurements would be required to determine whether and how the production of cytokines is being influenced by treatment, but such studies clearly are very difficult to achieve. Longitudinal analysis of intraocular fluids would also help to understand better the characteristics of pathogenesis during various stages of inflammation in IU. Analyses, standardized for specific uveitis entities and performed at similar time points during the course of the disease might identify a more specific cytokine profile and uncover the immunopathogenesis of diverse stages of intraocular inflammation.

Our patients with IU exhibited a consistent pattern of five highly-elevated immune mediators (IL-6, IL-8, IL-10, IL-12p70, and CCL2/MCP-1; each p<0.003). We cannot conclude that this combination is actually characteristic for IU because we lack data of other specific uveitis entities are lacking. However this pattern clearly differs from observations for FHUS, ocular Behçet disease or sarcoid uveitis [17,31-33].

In conclusion, our results reveal the intraocular production of multiple cytokines and chemokines during IU, some of which were linked to disease activity. High serum levels of TNF-α, independent of the presence of associated systemic disease, were typical, but, decreased under immunosuppressive treatment. The identification of proinflammatory molecules involved in the disease processes may lead to the development and implementation of new therapies. At present, TNF-α can be targeted, along with IL-6, IL-8, and CCL2/MCP-1 [34-36].

Our findings improve the understanding of the pathogenesis of IU and contribute to the identification of factors, which are involved in active IU.
